# Normothermic *ex vivo* heart perfusion with NLRP3 inflammasome inhibitor Mcc950 treatment improves cardiac function of circulatory death hearts after transplantation

**DOI:** 10.3389/fcvm.2023.1126391

**Published:** 2023-03-17

**Authors:** Liwei Xu, Zifeng Zeng, Chuanjie Niu, Deshen Liu, Shaoyan Lin, Xiu Liu, Gábor Szabó, Jun Lu, Shaoyi Zheng, Pengyu Zhou

**Affiliations:** ^1^ Department of Cardiovascular Surgery, Nanfang Hospital, Southern Medical University, Guangzhou, China; ^2^ Department of Cardiac Surgery, University of Heidelberg, Heidelberg, Germany; ^3^ Department of Cardiac Surgery, University of Halle (Saale), Halle, Germany

**Keywords:** heart transplantation, cardiovascular diseases, NLRP3, MCC950, donation after circulatory death, myocardial ischemia/reperfusion injury

## Abstract

**Background:**

The utilization of donation after circulatory death (DCD) hearts can enlarge the donor pool. However, DCD hearts suffer from serious ischemia/reperfusion injury (IRI). Recent studies found that the activation of NLRP3 inflammasome could play a significant role in organ IRI. Mcc950, which is a novel inhibitor of the NLRP3 inflammasome, can be applied to treat various kinds of cardiovascular diseases. Therefore, we hypothesized that the treatment of mcc950 could protect DCD hearts preserved with normothermic *ex vivo* heart perfusion (EVHP) against myocardial IRI *via* inhibiting NLRP3 inflammasome in a rat heart transplantation model of DCD.

**Methods:**

Donor-heart rats were randomly divided into four groups: Control group; Vehicle group; MP-mcc950 group; and MP + PO-mcc950 group. Mcc950 was added into the perfusate of normothermic EVHP in the MP-mcc950 and MP + PO-mcc950 groups, and was injected into the left external jugular vein after heart transplantation in the MP + PO-mcc950 group. Cardiac functional assessment was performed. The level of oxidative stress, inflammatory response, apoptosis, and NLRP3 inflammasome-associated protein of donor hearts were evaluated.

**Results:**

The treatment with mcc950 significantly increased the developed pressure (DP), dP/dt_max_, and dP/dt_min_ of the left ventricular of DCD hearts at 90 min after heart transplantation in both MP-mcc950 and MP + PO-mcc950 groups. Furthermore, mcc950 added into perfusate and injected after transplantation in both MP-mcc950 and MP + PO-mcc950 groups significantly attenuated the level of oxidative stress, inflammatory response, apoptosis, and NLRP3 inflammasome compared with the vehicle group.

**Conclusions:**

Normothermic EVHP combined with mcc950 treatment can be a promising and novel DCD heart preservation strategy, which can alleviate myocardial IRI *via* inhibiting NLRP3 inflammasome.

## 1. Introduction

Heart transplantation is recognized to be the ultimate treatment for patients with end-stage heart failure (1). However, the lack of suitable donor hearts obtained from brain-dead donor limits the development of heart transplantation (2). Nowadays, the utilization of donor hearts from donation after circulatory death (DCD) has been deemed to be an effective way to enlarge the donor pool (3).

Nevertheless, the inevitable warm ischemia time, which is from systolic blood pressure lower than 50 mmHg after the withdrawal of life-sustaining therapy to reperfusion or cardioplegia, results in more serious myocardial ischemia/reperfusion injury (IRI) in the DCD hearts, thereby leading to higher incidence of primary graft dysfunction (PGD) for the patients undergoing DCD heart transplantation (4, 5). Therefore, it's necessary to improve the current preservation strategy to reduce myocardial IRI of the DCD hearts.

Recent reports found that nucleotide-binding oligomerization domain-like receptor pyrin domain-containing 3 (NLRP3) inflammasome exerts a significant effect on increasing myocardial IRI (6, 7). Following the reperfusion after heart transplantation, the rapid generation of reactive oxygen species (ROS) can activate NLRP3 to bind to apoptosis-associated speck-like protein containing a caspase recruitment domain (ASC), thereby forming into the NLRP3 complex. The NLRP3 complex can combine with the effector caspase-1 and form the complete inflammasome, which can lead to the maturation of interleukin-1β (IL-1β) and IL-18 (8, 9), induce pro-inflammatory response, and finally aggravate myocardial IRI.

Mcc950 (IUPAC: N-[[(1,2,3,5,6,7-Hexahydro-s-indacen-4-yl)amino]carbonyl]-4-(1-hydroxy-1-methylethyl)-2-furansulfonamide sodium; Shorthand name: C_20_H_23_N_2_NaO_5_S; Molecular weight: 426.46), as a novel and selective inhibitor of NLRP3-inflammasome (10), interrupts the NLRP3-mediated ASC oligomerization, thereby preventing the activation of NLRP3 inflammasome. Recent studies demonstrated that mcc950 could significantly alleviate liver and kidney IRI during organ transplantation (11, 12). Mcc950, which is a novel inhibitor of the NLRP3 inflammasome, can be applied to treat various kinds of cardiovascular diseases, such as atherosclerosis and myocardial infarction (13–15). However, by far, there is no study investigating the protective effect of mcc950 against myocardial IRI within heart transplantation.

Recently, normothermic *ex vivo* heart perfusion (EVHP) is becoming a novel and promising preservation strategy for donor hearts (7). Compared with static cold storage which is a simple and traditional preservation method, normothermic EVHP can perfuse the beating donor hearts with warm, oxygenated and blood-based perfusate (16). Consequently, normothermic EVHP can significantly ameliorate myocardial IRI of DCD hearts, and surgeons can deliver preconditioning agents into the perfusion circuit to recondition the DCD hearts on this unique platform (17, 18).

Consequently, we hypothesized that the combination of normothermic EVHP and mcc950 treatment could ameliorate myocardial IRI and improve cardiac function of DCD hearts after heart transplantation. Therefore, in the present study, for the first time, we explored the cardioprotective effect of our well-established normothermic EVHP protocol (19) combined with mcc950 treatment in a rat DCD heart transplantation.

## 2. Materials and methods

### 2.1. Animals

Male Lewis rats (Charles River Laboratories, Beijing, China) used in this study received care in compliance with the Guide for the Care and Use of Laboratory Animals (National Institutes of Health Publication No. 85-23, revised 1996). All animal experiments were submitted, checked, and approved by the Ethical Committee of the Laboratory Animal Research Center of Southern Medical University Nanfang Hospital (animal protocol No. NFYY-2021-0243). The rats were housed in temperature-controlled (22 ± 2°C) rooms with a 12-h light-dark cycle, given food and sterilized water, and acclimatized for 1 week.

### 2.2. Experiment design

31 male Lewis rats (250–300 g; 10 to 12-week-old) were introduced as donor-heart rats and also blood donors for blood-based perfusate of the normothermic EVHP system. Another 31 male Lewis rats (300–350 g; 12 to 14-week-old) were regarded as recipient rats undergoing abdominal heterotopic heart transplantation.

In addition, another 6 male Lewis rats (250–300 g; 10 to 12-week-old) were introduced as donor-heart rats in the control group. In this control group, 6 male Lewis rats were directly sacrificed and their hearts tissue sample was collected.

**Figure 1:**
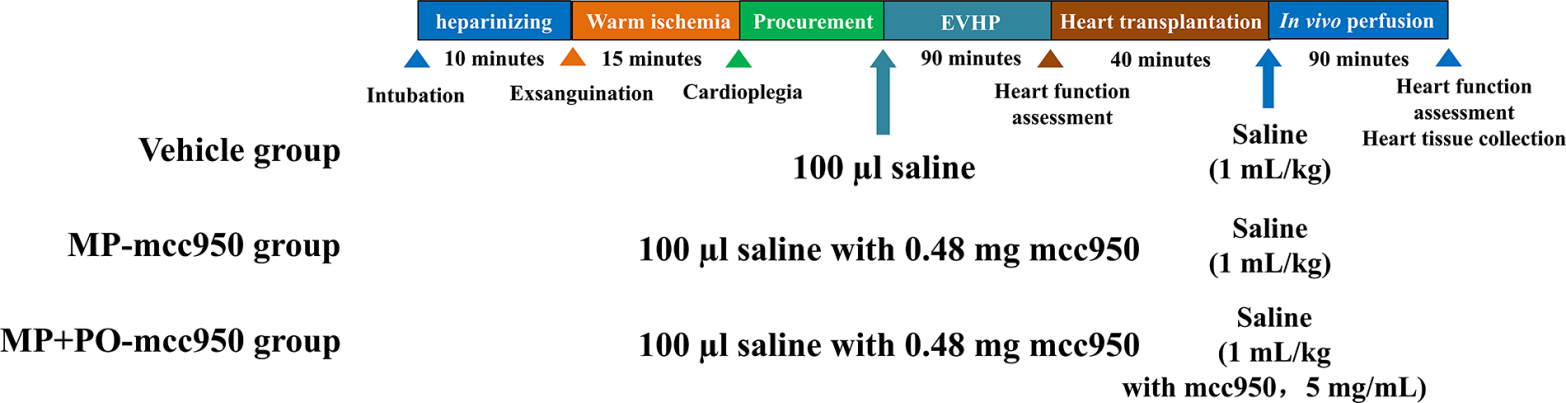
Study protocol of *ex vivo* heart perfusion combined with Mcc950 treatment in DCD heart preservation. DCD, donation after circulatory death; EVHP, *ex vivo* heart perfusion; MP, machine perfusion; PO, post-operation.

Except for the 6 rats in the control group, the other donor-heart rats were exsanguinated *via* the abdominal aorta to procure blood for EVHP. Subsequently, the donor-heart rats were subjected to the DCD procedure by suffering 15-min warm ischemia injury. Next, the hearts were procured and preserved by the normothermic EVHP system for 90 min. Cardiac function of the heart was assessed at the end of the normothermic EVHP period. Abdominal heterotopic heart transplantation was carried out after 90-min normothermic EVHP. At 90 min after heart transplantation, cardiac function of the donor heart was measured. Donor heart tissue and recipients' blood were collected to analyze the level of inflammation, apoptosis, oxidative stress, and NLRP3 inflammasome (Figure 1).

Donor-heart rats were divided into four groups randomly: (1) Control group: 6 non-DCD hearts were procured from heart-beating rats and heart tissue was immediately collected. (2) Vehicle group (11 rats): 100 µl saline was mixed into 12 ml blood-based perfusate before the initiation of normothermic EVHP and saline(1 ml/kg body weight) was injected into the left external jugular vein after heart transplantation; (3) MP (Machine perfusion)-mcc950 group (10 rats): 100 µl saline with mcc950 (Selleck, Houston, TX, USA; 0.48 mg) was mixed into 12 ml blood-based perfusate before the initiation of normothermic EVHP and saline (1 ml/kg body weight) was injected into the left external jugular vein after heart transplantation; (4) MP + PO (Post-operation)-mcc950 group (10 rats): 100 µl saline with mcc950 (Selleck, Houston, TX, USA; 0.48 mg) was added into 12 ml blood-based perfusate before the initiation of normothermic EVHP and mcc950 (1 ml/kg body weight, 5 mg/mL, dissolved in saline) was injected into the left external jugular vein after heart transplantation to achieve the dosage of 5 mg/kg body weight.

Two of the recipients were dead because of an overdose of anesthesia and another two recipients failed because of anastomotic leak. Finally, totally 31 pairs of rats underwent heart transplantation successfully and all of their donor hearts re-beat.

### 2.3. Operative procedure

**Figure 2:**
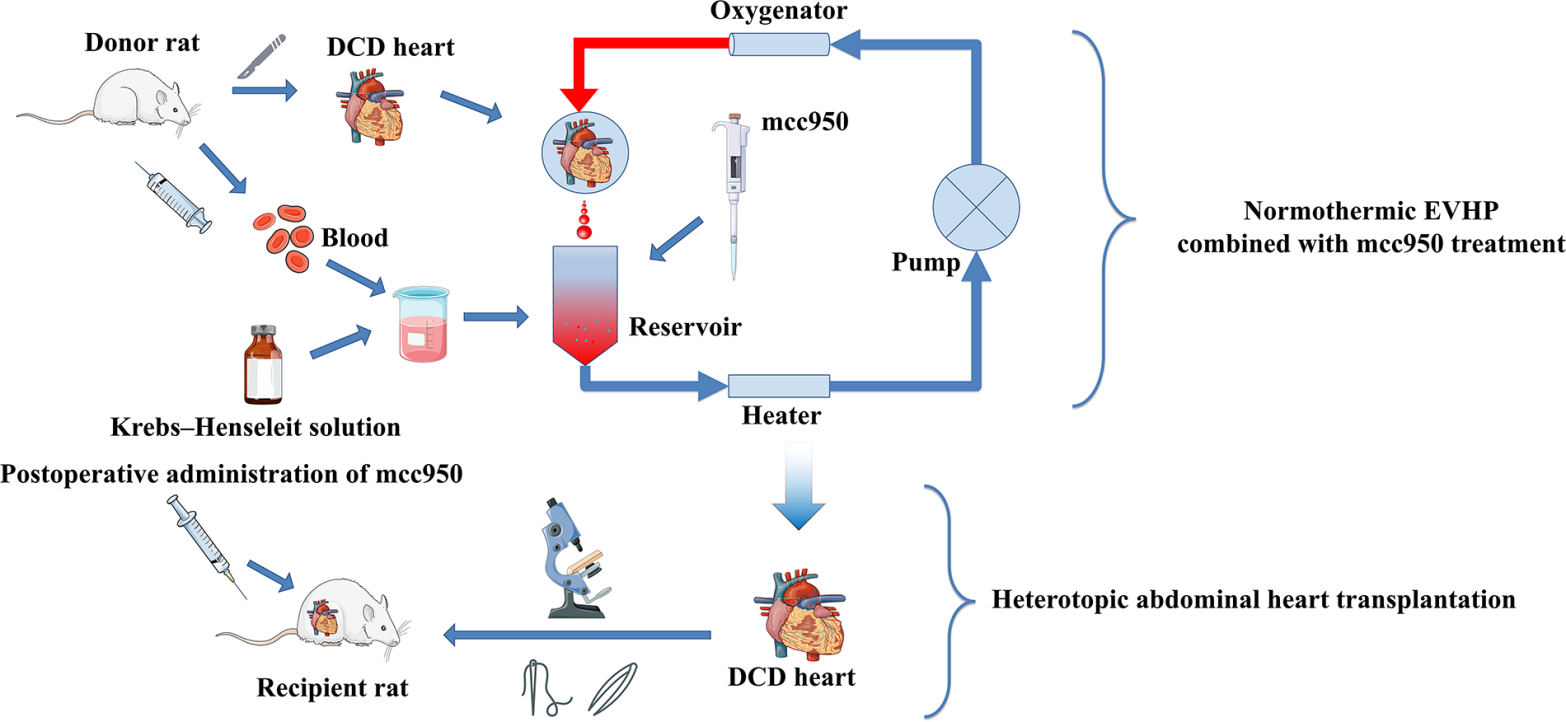
Schematic shows an overview of steps for normothermic EVHP and heterotopic abdominal heart transplantation. The donor rat was exsanguinated and its blood was collected to mix with Krebs–Henseleit solution to be the blood-based perfusate. After 15-min WIT, donor heart was procured and then perfused in the normothermic EVHP apparatus with mcc950. After 90-min EVHP, the donor heart was removed and heterotopic abdominal heart transplantation was performed by microsurgery. After a 10-min *in vivo* reperfusion, mcc950 was injected intravenously. EVHP, *ex vivo* heart perfusion.

Details of the operative procedure, including anesthesia, procuring of blood and donor heart from the donor rat, the operation of EVHP, heterotopic abdominal heart transplantation, and sample collection, are described in the Supplementary Material and briefly presented in Figure 2.

### 2.4. Analysis

#### 2.4.1. Oxidative stress

Immunohistochemistry was performed to analyze the expression of 4-hydroxynonenal (HNE), which is an indicator of oxidative stress in donor hearts. The immunoreactivity to HNE (1:1,000, Abcam, ab46545, United States) was measured. The antigen-antibody reaction was visualized by the diaminobenzidine reaction. The fields of each slice were selected and recorded randomly in a blinded manner under a conventional light microscope. Image-Pro Plus software (Media Cybernetics, United States) was applied to perform image analysis. Four random and non-overlapping fields of the heart tissue were selected for evaluation, and the average value of each rat was calculated. The expression of HNE was measured by counting integrated optical density (IOD).

#### 2.4.2. Inflammatory response

The expression of proinflammatory cytokines, including tumor necrosis factor-α (TNF-α), interleukin-6 (IL-6), and nuclear factor kappa-B p65 (NF-*κ*B p65) in myocardial tissue were measured by western blotting as described below.

#### 2.4.3. Apoptosis

Terminal deoxynucleotidyl transferase dUTP nick end labeling (TUNEL) staining (KeyGEN BioTECH, KGA7073, China) was performed to detect DNA-strand breaks of the donor hearts as previously described (20). TUNEL-positive cells were counted by using a fluorescence microscope and the frequency of apoptosis in the donor heart was defined as the ratio of 40,6-diamidino-2-phenylindole (DAPI)-TUNEL double-labeled nuclei to the total number of nuclei stained with DAPI.

#### 2.4.4. Immunofluorescence

With regard to the immunofluorescence method, myocardial tissue slices were incubated with the following primary antibodies: Rabbit anti-NLRP3 (1:100, Proteintech, 19771-1-AP, United States), anti-Caspase 1 (1:50, Proteintech, 22915-1-AP, United States), anti-IL-1β (1:200, Proteintech, 26048-1-AP, United States), anti-IL-18 (1:400, Proteintech, 10663-1-AP, China), anti-ASC (1:800, Cell Signaling, #67824, United States).

The slices were then washed and detected with appropriate Fluor dye, DyLight 488 (1:50, EarthOx, E032220-01, United States), as secondary antibodies followed by counterstaining with DAPI in the dark. Afterward, immunofluorescent signaling was observed with a fluorescence microscope (IX73 Olympus). Digital images and data were recorded and analyzed by applying ImageJ (NIH).

#### 2.4.5. Western blotting

Myocardial protein expression of donor hearts was assessed as previously described by western blotting (21). The expression of Caspase-3 [1:1,000 dilution, #14220, Cell Signaling Technology (Shanghai) Biological Reagents Company Limited, China], ASC [1:1,000 dilution, #67824, Cell Signaling Technology (Shanghai) Biological Reagents Company Limited, China], Caspase-1 (1:1,000 dilution, 22915-1-AP), Bax (1:5,000 dilution, 60267-1-Ig), Bcl-2 (1:1,000 dilution, 60178-1-Ig), NLRP3 (1:1,000 dilution, 19771-1-AP), IL-1β (1:1,000 dilution, 26048-1-AP), IL-18 (1:1,000 dilution, 10663-1-AP), IL-6 (1:1,000 dilution, 23457-1-AP), NF-*κ*B (1:1,000 dilution, 66535-1-Ig), and TNF-α (1:1,000 dilution, 19590-1-AP) were evaluated. Except for Caspase-3 and ASC, the other reagents were all purchased from Proteintech Group, Inc, USA.

### 2.5. Statistical analysis

The results were expressed as mean ± standard error of the mean (SEM). GraphPad Prism 8.3 software (GraphPad Sofware, Inc., San Diego, CA, United States) was used to perform statistical analysis. Shapiro-Wilk test was performed to test the normality of data before statistical tests were applied. One-way ANOVA followed by Tukey's post-hoc-test was performed for multiple comparisons between three experimental groups. If the data failed the normality test, the non-parametric Kruskal–Wallis test followed by Dunn's post-hoc-test was used. Intraventricular pressure measurement recordings among vehicle, MP-mcc950, and MP + PO-mcc950 groups were compared by two-way ANOVA according to different time points. A value of *p* < 0.05 was considered statistically significant.

## 3. Results

### 3.1. The NLRP3 inflammasome is involved in the warm ischemia-reperfusion injury for the DCD hearts

To test a hypothesis that NLRP3 inflammasome could be presented in the DCD hearts which had suffered myocardial IRI, the expression of NLRP3, ASC, Caspase-1, IL-1β and IL-18 in the donor hearts was evaluated by immunofluorescence and western blotting analysis.

**Figure 3:**
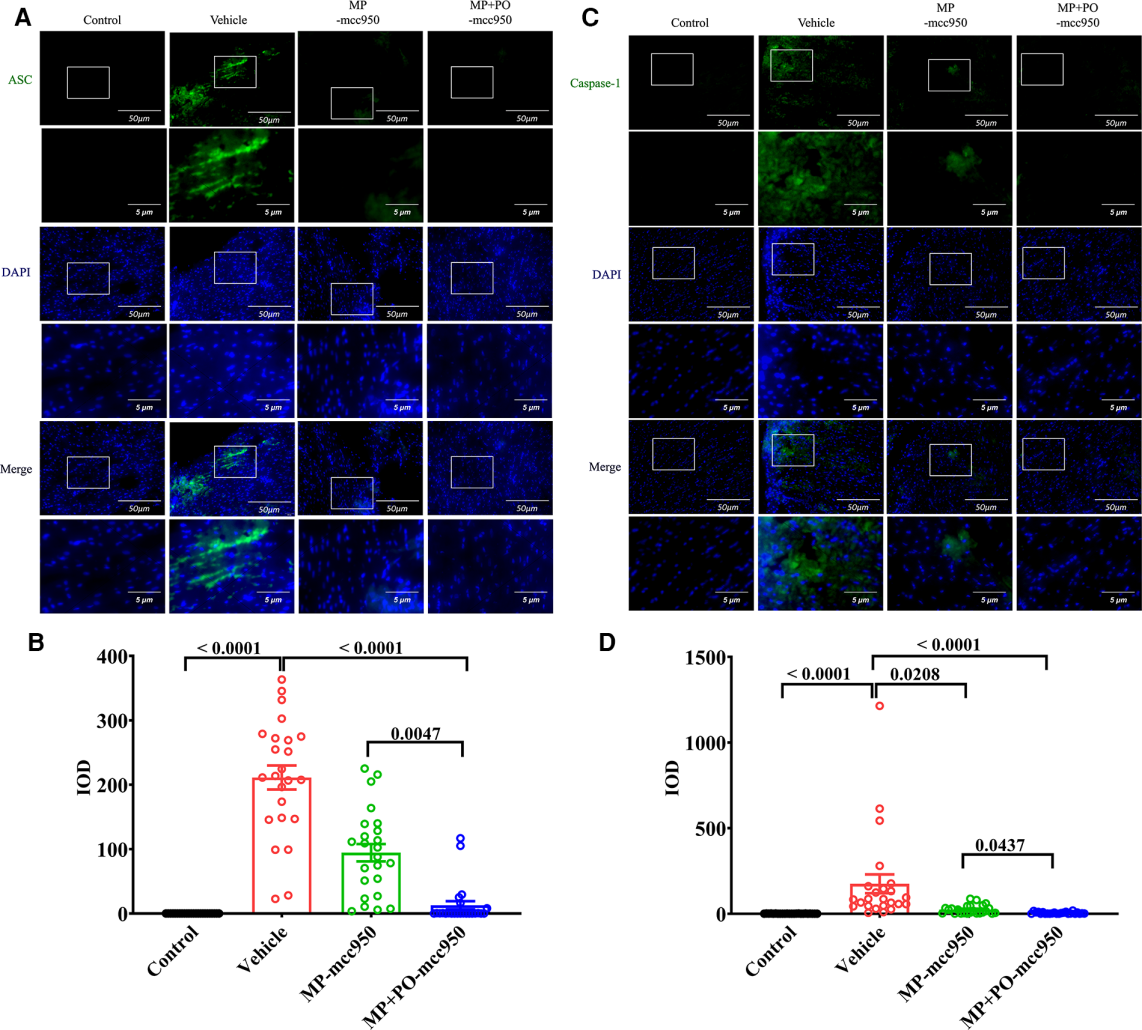
Representative immunofluorescence staining of NLRP3 inflammasome-associated protein ASC and caspase-1 in the hearts. Representative immunofluorescence staining images of (**A**) ASC and (**C**) Caspase-1 in myocardial tissue (green). 4′,6-diamidino-2-phenylindole (DAPI) was used for nuclear counterstaining (blue), scale bar 50 μm. The magnification of the images was shown below, scale bar 5 μm. Quantitative analysis of immunohistochemistry for (**B**) ASC and (**D**) Caspase-1 among the four groups was shown below respectively. Data represent mean ± standard error of the mean. *n* = 6 for each group. The *p*-values are shown above. MP, machine perfusion; PO, post-operation; NLRP3, nucleotide-binding oligomerization domain-like receptor family pyrin domain-containing 3; ASC, apoptosis-associated speck-like protein containing CARD; IOD, integrated optical density.

**Figure 4:**
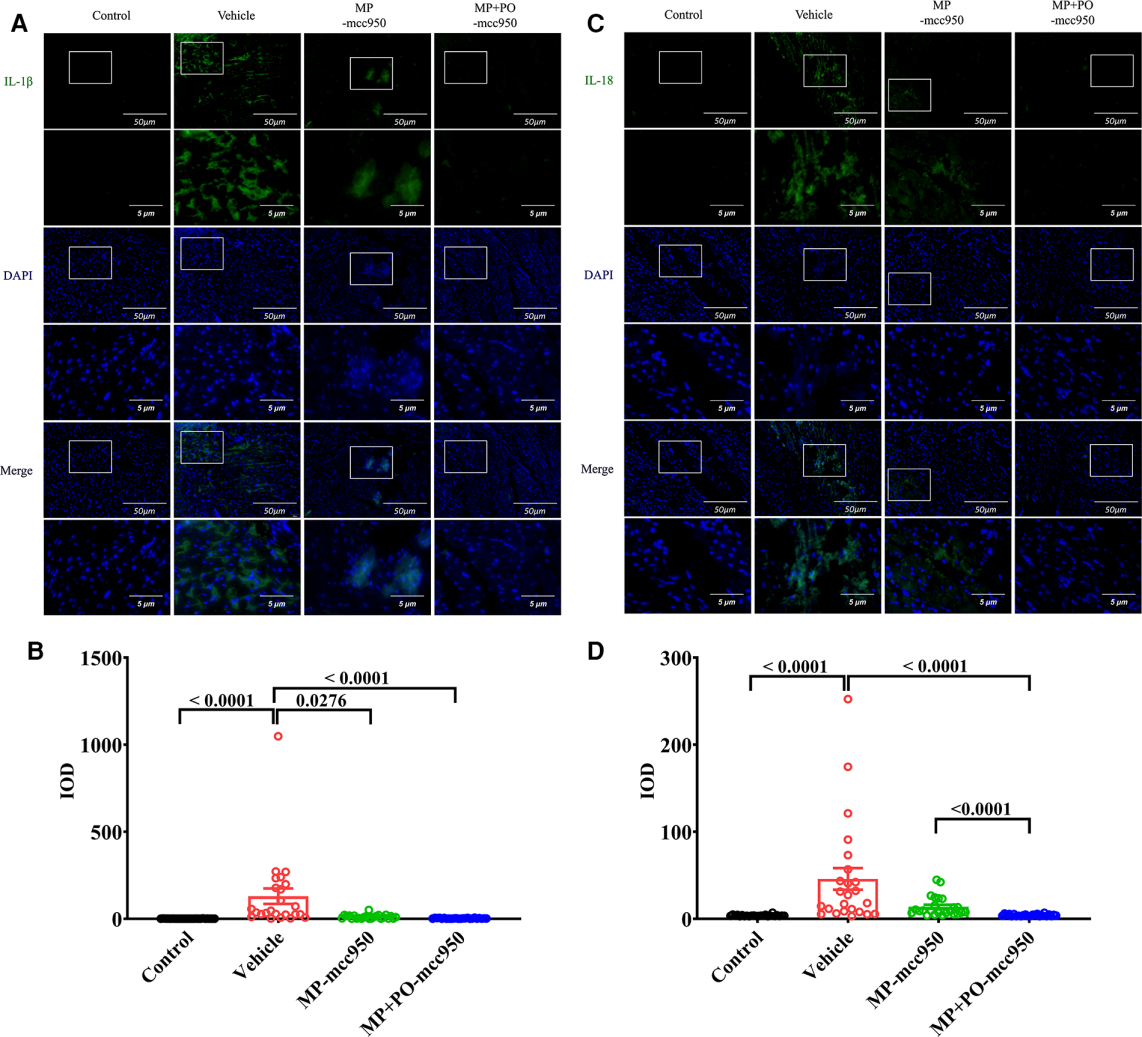
Representative immunofluorescence staining of NLRP3 inflammasome-associated protein IL-1β and IL-18 in the hearts. Representative immunofluorescence staining images of (**A**) IL-1β and (**C**) IL-18 in myocardial tissue (green). 4′,6-diamidino-2-phenylindole (DAPI) was used for nuclear counterstaining (blue), scale bar 50 μm. The magnification of the images was shown below, scale bar 5 μm. Quantitative analysis of immunohistochemistry for (**B**) IL-1β, and (**C**) IL-18 among the four groups was shown below respectively. Data represent mean ± standard error of the mean. *n* = 6 for each group. The *p*-values are shown above. MP, machine perfusion; PO, post-operation; IL-1β, interleukin-1β; IL-18, interleukin-18; IOD, integrated optical density.

**Figure 5:**
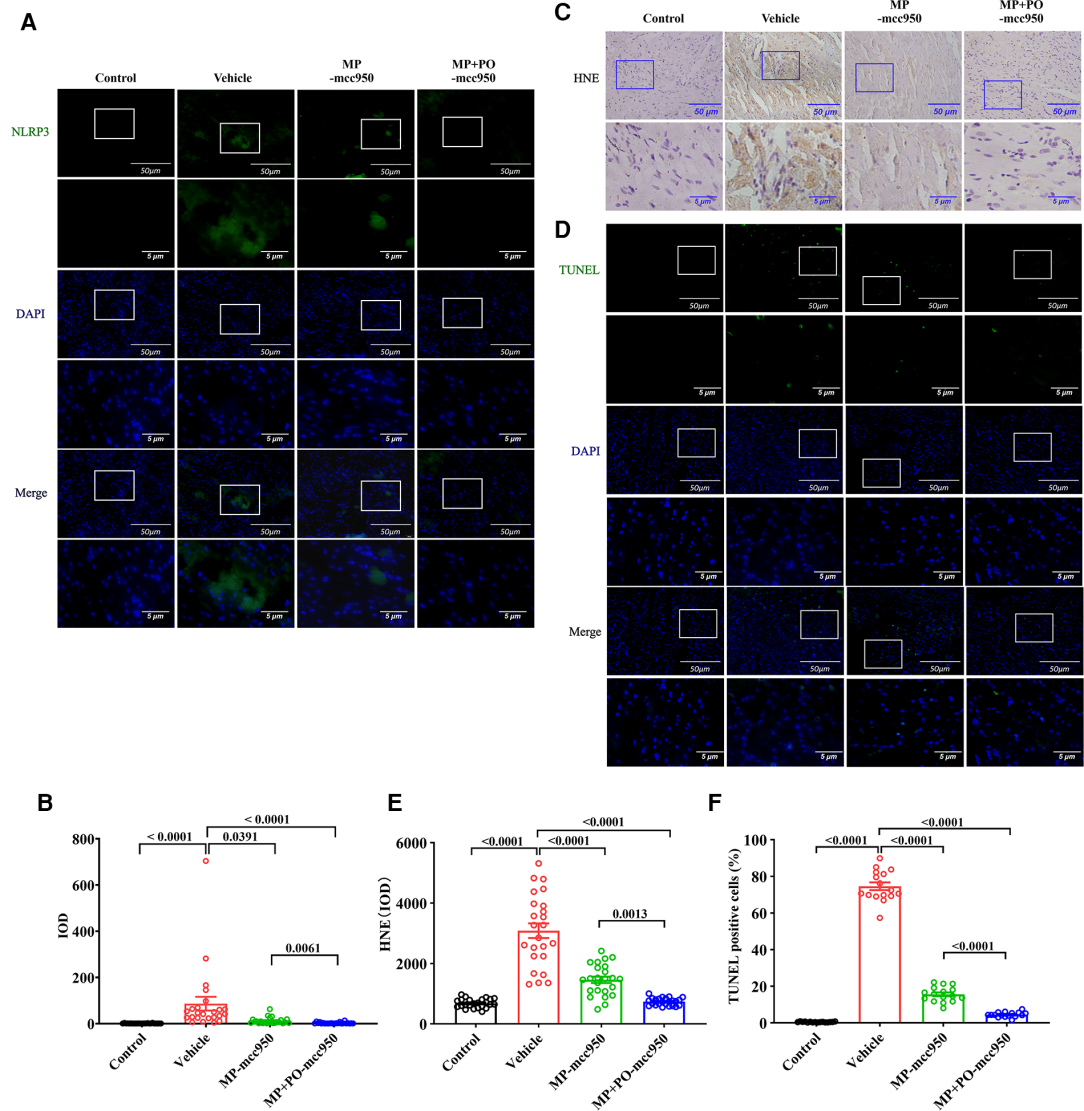
Representative immunofluorescence staining of NLRP3 inflammasome-associated protein NLRP3 in the hearts and *ex vivo* heart perfusion with mcc950 treatment ameliorates myocardial oxidative stress and apoptosis in the DCD hearts. (**A**) Representative immunofluorescence staining images of NLRP3 in myocardial tissue (green). 4′,6-diamidino-2-phenylindole (DAPI) was used for nuclear counterstaining (blue), scale bar 50 μm. The magnification of the images was shown below, scale bar 5 μm. (**B**) Quantitative analysis of immunohistochemistry for NLRP3 among the four groups was shown below respectively. Data represent mean ± standard error of the mean. *n* = 6 for each group. (**C**) Representative photomicrographs and quantitative analysis of immunohistochemistry for HNE; (**D**) Representative photomicrographs of myocardial tissue stained with DAPI (blue), nuclei with fragmented DNA shown by TUNEL staining (green), and merged image (scale length: 50 µm). The magnification of the images was shown below, scale bar 5 μm. (**E**) Quantitative analysis of immunohistochemistry for HNE in the DCD heart. Data represent mean ± standard error of the mean. *n* = 6 for each group. (**F**) Quantification of TUNEL-positive cells (as a percentage). Data represent mean ± standard error of the mean. *n* = 4 for each group. DAPI, 40,6-diamino-2-phenylindole (DAPI, blue); TUNEL, terminal deoxynucleotidyl transferase-mediated dUTP nick end-labeling. The *p*-values are shown above. MP, machine perfusion; PO, post-operation; HNE, 4-hydroxynonenal; IOD, integrated optical density.

**Figure 6:**
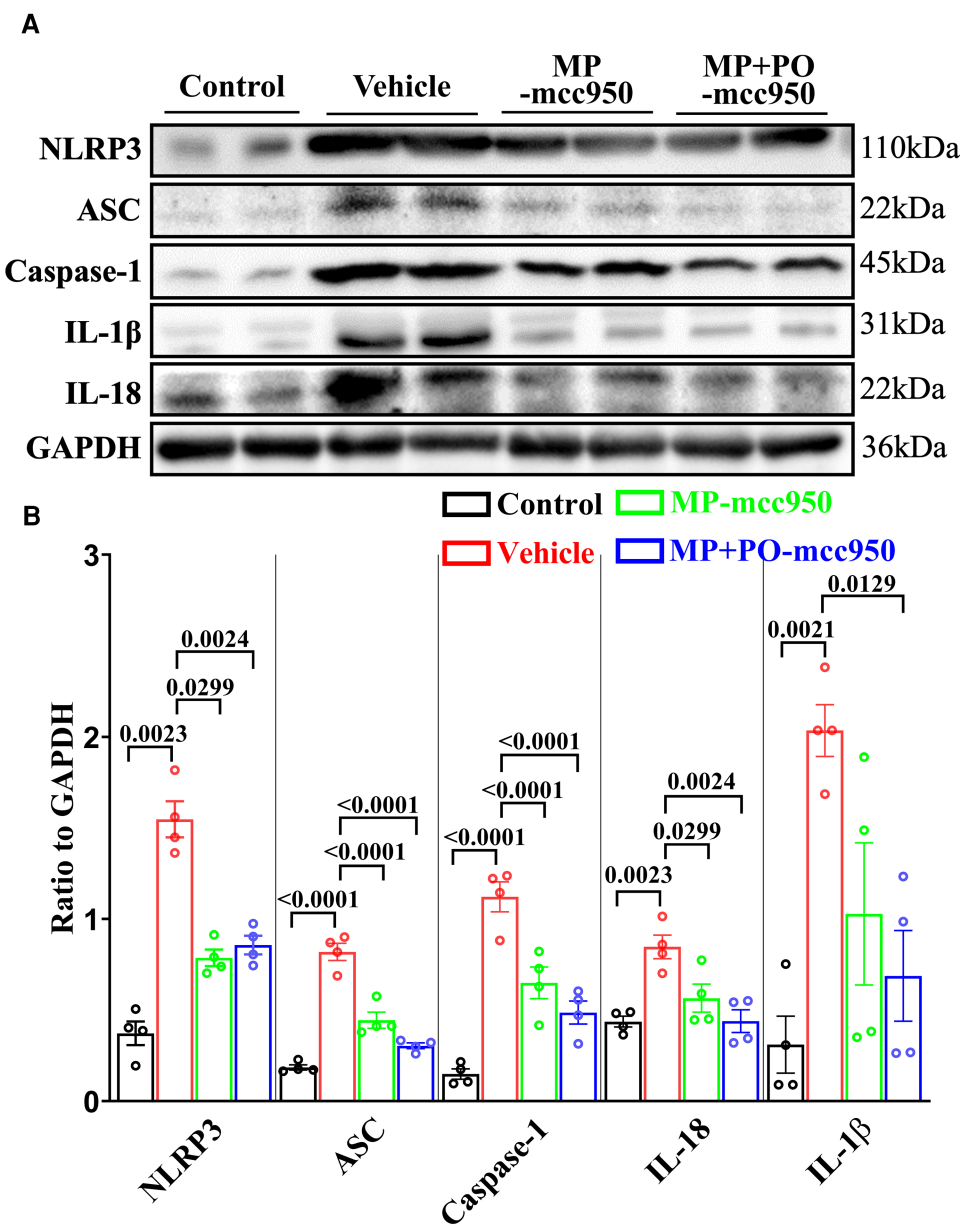
Representative western blotting and quantitative analysis of the effect of mcc950 treatment on NLRP3 inflammasome in donor hearts. (**A**) Representative protein band densities of NLRP3 inflammasome associated proteins; (**B**) Quantitative analysis of protein expression. Data represent mean ± standard error of the mean. *n* = 4 for each group. The *p*-values are shown above. MP, machine perfusion; PO, post-operation; NLRP3, nucleotide-binding oligomerization domain-like receptor family pyrin domain-containing 3; ASC, apoptosis-associated speck-like protein containing CARD; IL-1β, interleukin-1β; IL-18, interleukin-18.

In (Figures 3–5A,B), compared with the control group, the expression level of NLRP3, ASC, Caspase-1, IL-1β, and IL-18 significantly increased in the vehicle group, as presented by immunofluorescence analysis. Moreover, the expression level of the NLRP3 inflammasome-associated protein in the vehicle group significantly increased compared with the control group, as indicated by the western blotting analysis (Figure 6).

### 3.2. Ex vivo heart perfusion with Mcc950 treatment improves cardiac function impairment caused by warm ischemia-reperfusion injury in the DCD hearts

**Figure 7:**
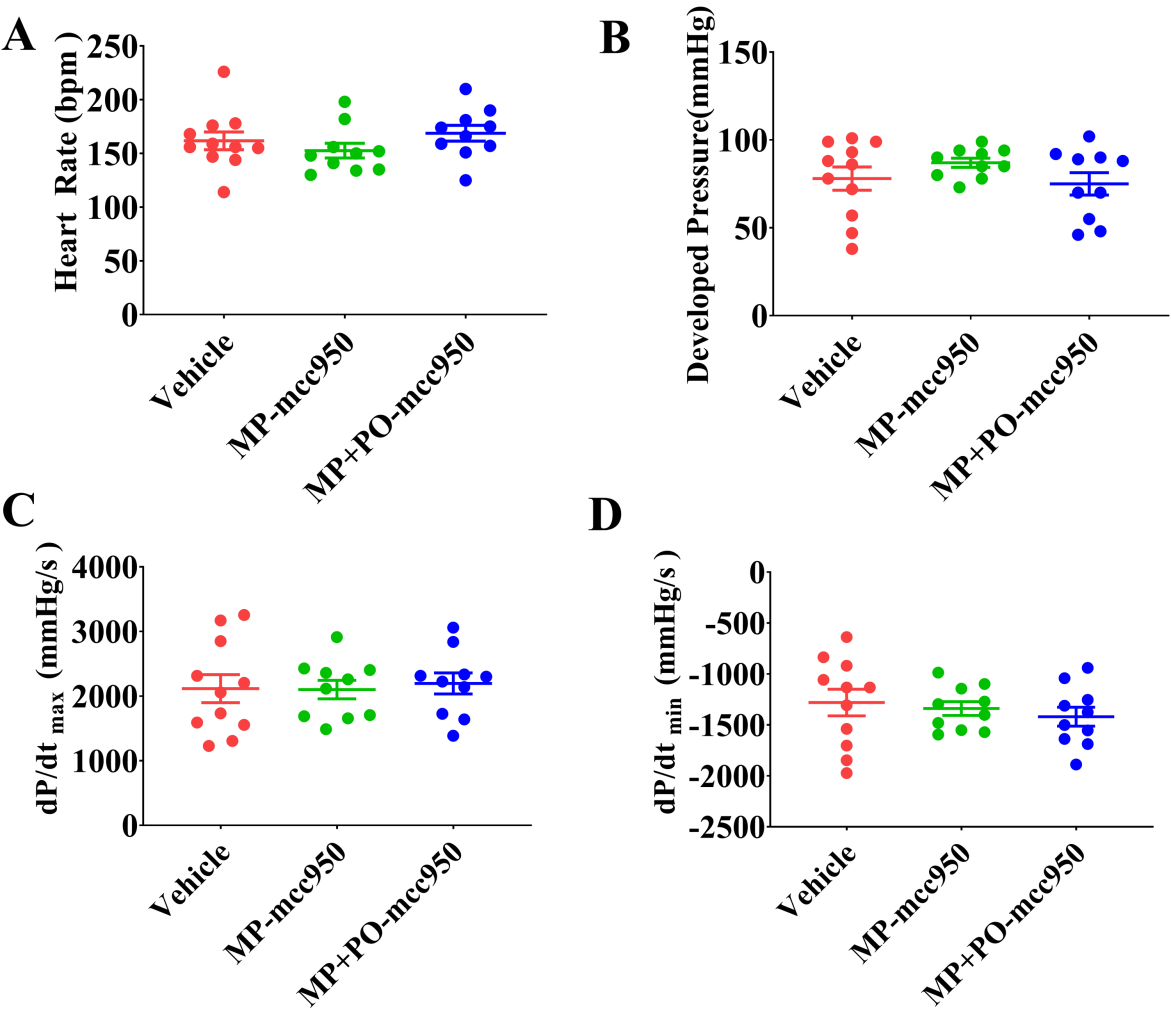
The effect of *ex vivo* heart perfusion with mcc950 treatment on the cardiac function of the DCD hearts at the end of EVHP. (**A**) Heart rate; (**B**) developed pressure; (**C**) dP/dt_max_; and (**D**) dP/dt_min_. Data represent mean ± standard error of the mean. *n* = 11 or 10 for each group. Two-way ANOVA was applied in statistical analysis. MP, machine perfusion; PO, post-operation; dP/dt_max_ (maximum rate of rise of left ventricular pressure), and dP/dt_min_ (maximum rate of pressure decline).

As shown in Figure 7, there was no significant difference among the three groups with regard to the cardiac function of donor hearts measured at the end of 90-min normothermic EVHP.

**Figure 8:**
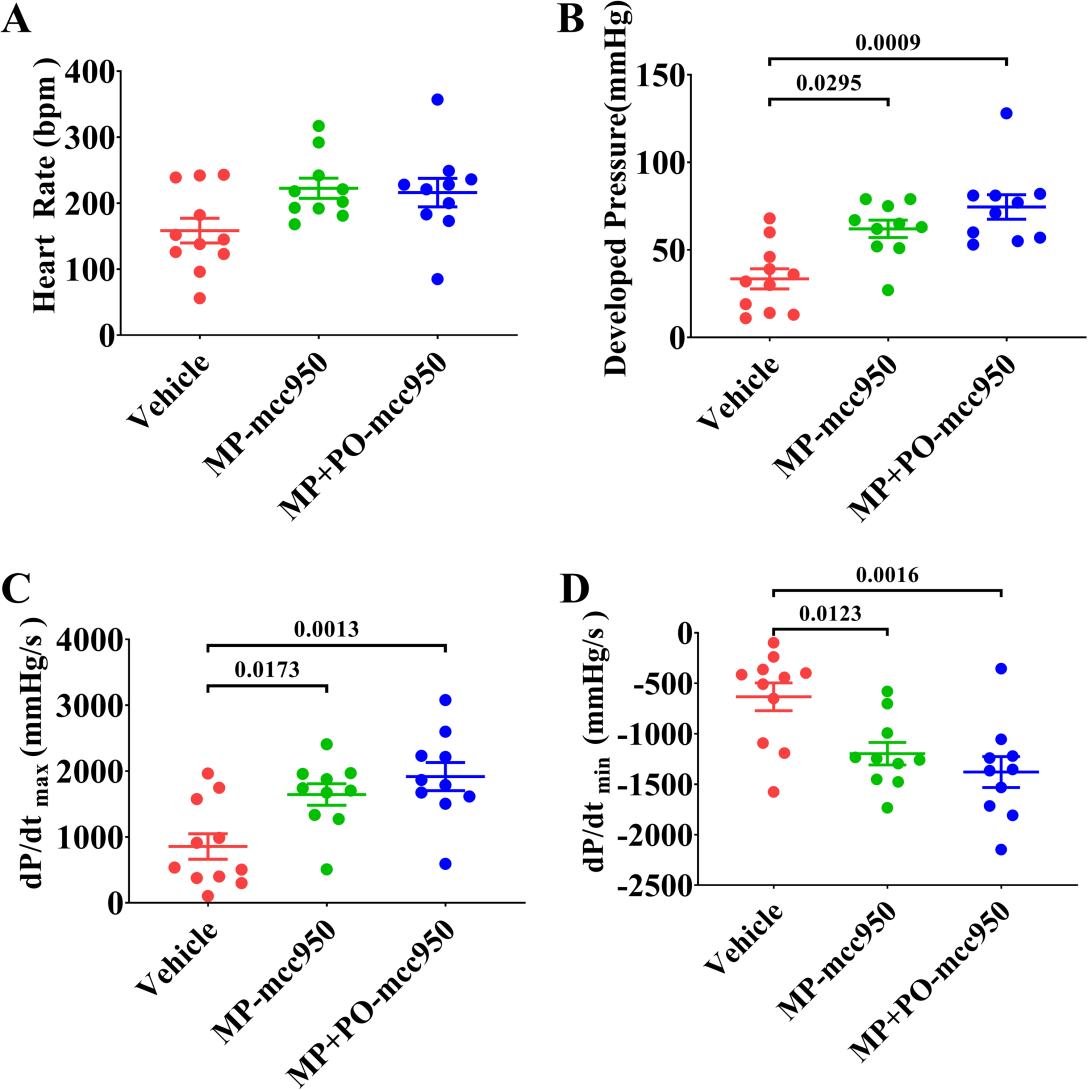
*Ex vivo* heart perfusion with mcc950 treatment improves the post-transplant cardiac function of the DCD hearts. Cardiac function was measured 90 min after heart transplantation. (**A**) Heart rate; (**B**) developed pressure; (**C**) dP/dt_max_; and (**D**) dP/dt_min_. Data represent mean ± standard error of the mean. *n* = 11 or 10 for each group. Two-way ANOVA was applied in statistical analysis. The adjusted *p*-values are shown. MP, machine perfusion; PO, post-operation; dP/dt_max_ (maximum rate of rise of left ventricular pressure), and dP/dt_min_ (maximum rate of pressure decline).

We measured the cardiac function of donor hearts 90 min after heart transplantation. As indicated in Figure 8, compared with the vehicle group, there was a significant increase in the cardiac function of donor hearts at 90 min after heart transplantation, including developed pressure, dP/dt_max,_ and dP/dt_min_, in the MP-mcc950 and MP + PO-mcc950 groups. However, no significant difference could be observed among the three groups concerning heart rate. Furthermore, there was no significant difference between the MP + PO-mcc950 and MP-mcc950 groups in terms of post-transplant cardiac function of donor hearts.

### 3.3. Ex vivo heart perfusion with Mcc950 treatment attenuates oxidative stress in the DCD hearts

As an indicator of oxidative stress, the expression of HNE in the donor hearts among the four groups was determined by immunohistochemical analysis. Compared with the control group, warm ischemia-reperfusion injury significantly increased the expression of HNE in the vehicle group. However, the addition of mcc950 into the circuit of EVHP could lead to a significantly decreased expression of HNE in the DCD hearts 90 min after heart transplantation. Furthermore, the additional administration of mcc950 after heart transplantation in the MP +PO-mcc950 group could significantly reduce the expression of HNE in the donor hearts compared with the MP-mcc950 group (Figures 5C,E).

### 3.4. Ex vivo heart perfusion with Mcc950 treatment ameliorates myocardial apoptosis in the DCD hearts

A significantly increased number of TUNEL-positive nuclei in the heart graft could be observed in the vehicle group compared with the control group. However, the treatment with mcc950 during normothermic EVHP in the MP-mcc950 group significantly decreased DNA strand breaks in the DCD hearts after heart transplantation compared with the vehicle group. Finally, the level of myocardial apoptosis in the DCD hearts was significantly lowered in the MP + PO-mcc950 group compared with the MP-mcc950 group (Figures 5D,F).

**Figure 9:**
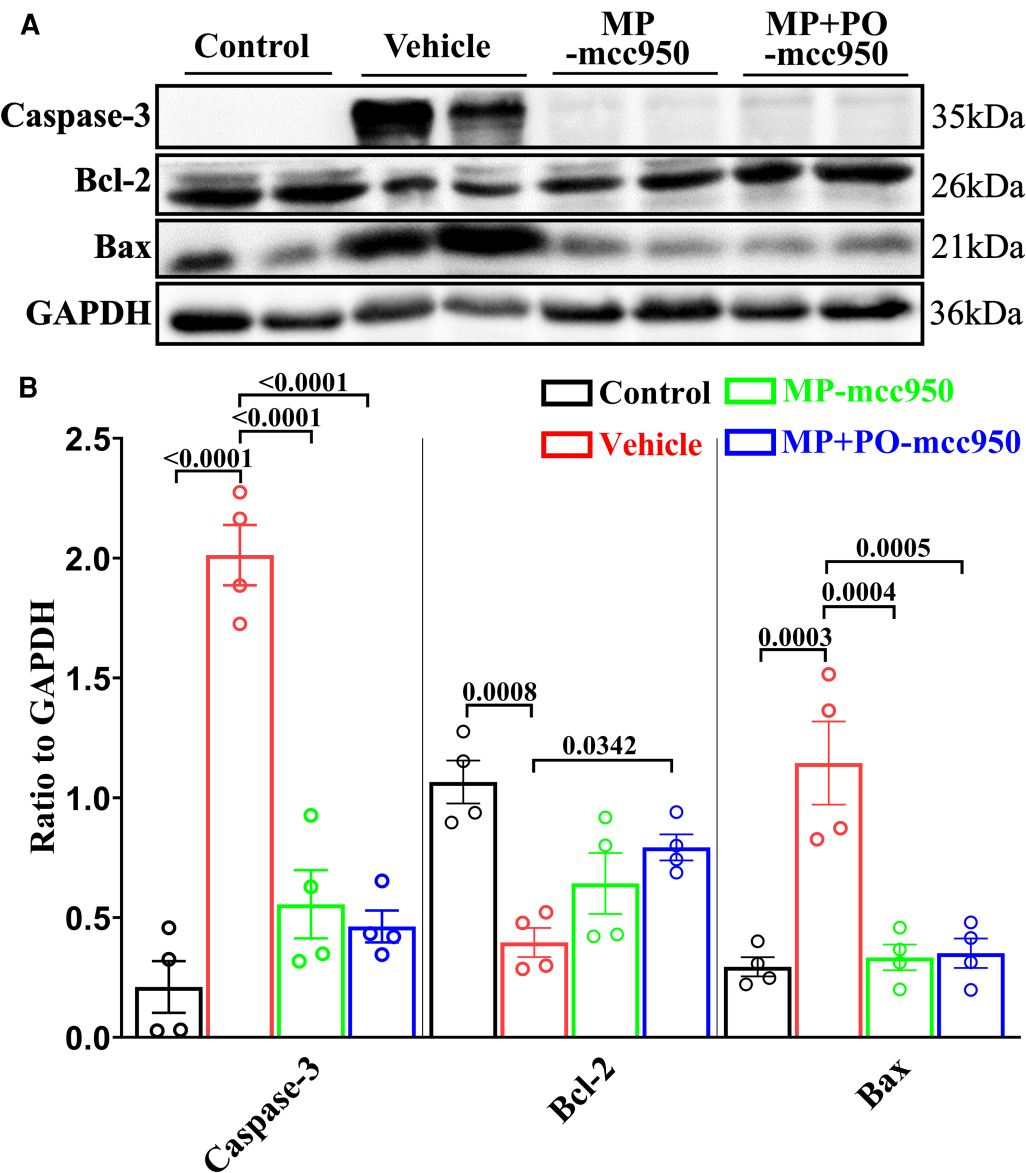
Representative western blotting and quantitative analysis of the effect of mcc950 treatment on apoptosis in the DCD hearts preserved with EVHP. (**A**) Representative protein band densities of apoptosis-associated proteins; (**B**) Quantitative analysis of protein expression. Data represent mean ± standard error of the mean. *n* = 4 for each group. The *p*-values are shown above. MP, machine perfusion; PO, post-operation; Bcl-2, B-cell lymphoma-2; Bax, Bcl-2-Associated X.

In addition, western blot assay showed that the expression of pro-apoptosis protein markers Bax and Caspase-3 significantly decreased, while anti-apoptosis protein Bcl-2 increased in the MP + PO-mcc950 groups compared with the vehicle group (Figure 9).

### 3.5. Ex vivo heart perfusion with Mcc950 treatment attenuates inflammation in the DCD hearts

**Figure 10:**
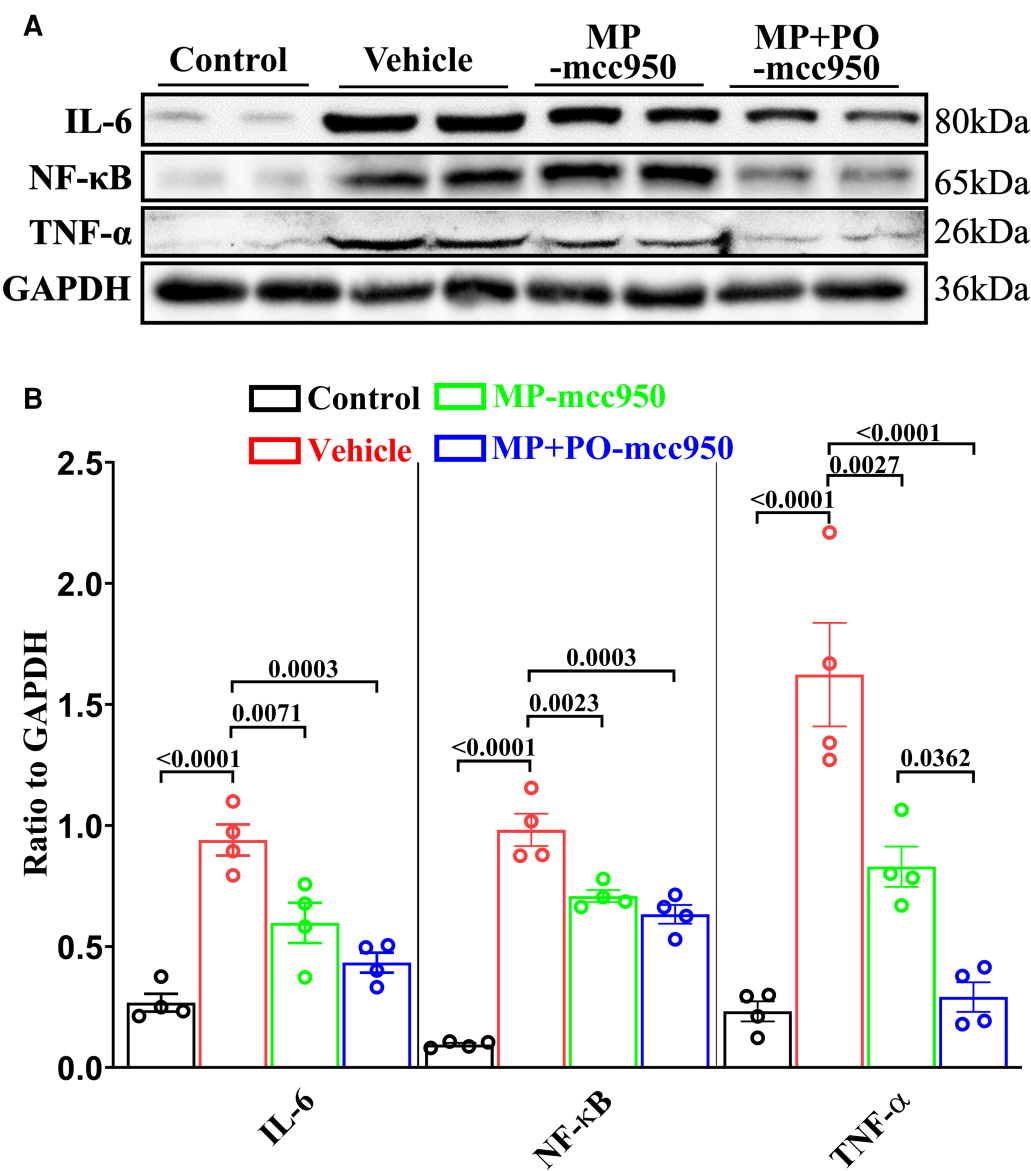
Representative western blotting and quantitative analysis of the effect of mcc950 treatment on inflammatory response in donor hearts preserved with EVHP. (**A**) Representative protein band densities of inflammatory response associated proteins; (**B**) Quantitative analysis of protein expression. Data represent mean ± standard error of the mean. *n* = 4 for each group. The *p*-values are shown above. MP, machine perfusion; PO, post-operation; IL-6, interleukin-6; TNF-α, tumor necrosis factor-α; NF-*κ*B, nuclear factor kappa-B.

The expression of IL-6, TNF-α, and NF-*κ*B in heart tissue was measured by western blotting to evaluate the inflammatory response in the myocardium. As shown in Figure 10, a WIT of 15 min significantly up-regulated the levels of IL-6, TNF-α and NF-*κ*B in the DCD heart submitted to EVHP, while the treatment of mcc950 significantly attenuated inflammatory response in the DCD heart in MP-mcc950 and MP + PO-mcc950 groups.

### 3.6. Ex vivo heart perfusion with Mcc950 treatment inhibits NLRP3 inflammasome generation in the DCD hearts

The expression of NLRP3, ASC, Caspase-1, IL-1β, and IL-18 in the donor hearts, which demonstrates the generation of the NLRP3 inflammasome, was evaluated by immunofluorescence analysis and western blotting.

In the immunofluorescence analysis, compared with the vehicle group, the level of the NLRP3 inflammasome significantly reduced in the post-transplant donor hearts in the MP-mcc950 and MP + PO-mcc950 groups (Figures 3–5). In addition, as shown in the result of western blotting, compared with the vehicle group, the level of the NLRP3 inflammasome significantly decreased in the DCD hearts of the MP-mcc950 and MP + PO-mcc950 groups (Figure 6).

## 4. Discussion

In our present study, we demonstrated the protective effect of the selective NLRP3 inflammasome inhibitor mcc950 on transplantation outcomes in a rat DCD heart transplantation model. *Ex vivo* heart perfusion with mcc950 treatment could protect the DCD hearts against myocardial IRI by attenuating the level of NLRP3 inflammasome.

Increasing evidence showed that NLRP3 inflammasome was a potential therapeutic target for IRI in myocardial infarction (22, 23). Shen et al. (24) also reported that NLRP3 inflammasome could participate in myocardial IRI and NLRP3-mediated pyroptosis. Raffaella et al. (25) discovered the crucial role of NLRP3 inflammasome activation in the early step of myocardial injury caused by ischemia and reperfusion. Moreover, the pharmacological inhibition of NLRP3 inflammasome, INF4E, could reduce the level of NLRP3 and inhibit the downstream activation of Caspase-1, GSDMDC1, and IL-1β cleavage on the rat hearts, thereby relieving the myocardial injury and cardiac dysfunction.

In line with these previous studies, we also observed that the level of NLRP3 inflammasome (as shown by the expression of NLRP3, ASC, cleaved caspase-1, IL-1β, IL-18) in the vehicle group significantly increased compared with the control group. Therefore, inhibiting the activation of NLRP3 inflammasome could be a significant therapeutic target in reducing myocardial IRI in DCD hearts. In line with this novel theory, our previous study (19), demonstrated EVHP combined with melatonin preconditioning attenuated myocardial IRI in DCD hearts by inhibiting NLRP3 inflammasome-mediated pyroptosis. However, the cardioprotective effect of normothermic EVHP combined with melatonin on the post-transplant donor heart remained unclear in this study due to the lack of a heart transplantation model.

Mcc950, as a selective inhibitor, has been shown to inhibit the activation of the NLRP3 inflammasome and expression of Caspase-1, IL-18, and IL-1β. Yu et al. (11) reported that the outcome of DCD liver could be improved by the addition of mcc950 to the perfusate of the hypothermic machine perfusion system and intravenous injection of mcc950 after liver transplantation. Zheng et al. (26) proved that mcc950 mitigated post-resuscitation myocardial dysfunction and improved rat survival by reducing NLRP3 inflammasome. In addition, Zhang et al. (27) showed mcc950 attenuated doxorubicin-induced myocardial injury *in vivo* and *in vitro* by inhibiting NLRP3-mediated pyroptosis. Consequently, we hypothesized that normothermic EVHP and mcc950 treatment could be a novel and effective organ preservation strategy, which might reduce myocardial IRI in the DCD hearts and increase the number of transplantable hearts. In the present study, we introduced the heterotopic heart transplantation model to fully present the cardioprotective potential of normothermic EVHP combined with mcc950 treatment. Cardiac function, as reflected by developed pressure, dP/dt_max_, and dP/dt_min_, significantly improved in the DCD hearts in the MP-mcc950 and MP + PO-mcc950 group at 90 min after heart transplantation. However, no significant difference could be found in the cardiac function of DCD hearts between the MP + PO-mcc950 and MP-mcc950 groups. We supposed that the treatment of mcc950 after transplantation might influence the intracellular metabolism of myocardial tissue, including the generation of ROS and apoptosis (as shown by the expression of HNE and number of TUNEL-positive nuclei, respectively), but not the cardiac function of DCD hearts. As Anantha et al. (28) showed, the activation of IL-18 and IL-1β inflammatory cytokines could induce the release of cell contents, and finally triggered ROS-inflammasome-mediated inflammatory cascade.

Apoptosis, as one kind of programmed cell death, was reported to be suppressed when NLRP3 inflammasome was inhibited (19, 29). The inhibition of NLRP3 inflammasome can lead to the decreased expression of IL-1β (30). Consequently, the IL-1β-induced apoptosis can be alleviated through NF-*κ*B, MAPKs and PI3K/Akt signaling pathway (31). In our previous study (16), we also found melatonin inhibited NLRP3 inflammasome to relieve apoptosis in DCD hearts. In the current study, by measuring the expression of Bax, Bcl-2, Caspase-3, and the number of TUNEL-positive nuclei, we can conclude that warm ischemia-reperfusion injury leads to myocardial apoptosis in the DCD hearts, while mcc950 treatment attenuates NLRP3 inflammasome-mediated apoptosis (32).

In the present study, no significant difference was observed among vehicle and MP-mcc950 groups with regard to the cardiac function of DCD hearts measured at the end of 90-min normothermic EVHP. Furthermore, there was no significant difference between MP + PO-mcc950 and MP-mcc950 groups in terms of post-transplant cardiac function of donor hearts measured at the end of 90-min reperfusion. We supposed that 90-min reperfusion, either EVHP or heterotopic abdominal heart transplantation, might not be long enough for mcc950 to significantly exert its cardioprotective effect on DCD hearts. However, mcc950 preconditioning during 90-min EVHP could significantly improve the post-transplant cardiac function of DCD hearts, which indicated that 180-min reperfusion, including 90-min EVHP and 90-min reperfusion after heterotopic heart transplantation, could be an optimal timepoint for us to observe the profoundly improved cardiac function as a result of mcc950 treatment.

There are several limitations of this study that need to be discussed. Firstly, the induction of circulatory death in the rats was exsanguination, which might not closely mimic a clinical DCD setting. Secondly, the dosage of mcc950 in the perfusate for normothermic EVHP might not be ideal. Compared with the previous study (11) in which they adopted hypothermic machine perfusion to preserve pig livers, we applied normothermic machine perfusion to preserve rat hearts with a high metabolic rate in the present study. Consequently, we chose the same dosage as the reference. (40 mg/l). Finally, we only investigated the short-term (90 min) post-transplant graft function of DCD hearts. Consequently, it is necessary to conduct future transplant studies to evaluate the long-term post-transplant cardiac function of DCD hearts.

## 5. Conclusion

The present study reveals that normothermic EVHP combined with mcc950 treatment can alleviate myocardial IRI *via* inhibiting NLRP3 inflammasome in a rat heart transplantation model of DCD. Therefore, normothermic EVHP combined with mcc950 treatment could be a promising and novel DCD heart preservation strategy, thereby increasing the number of transplantable hearts in heart transplantation.

## Data Availability

The raw data supporting the conclusions of this article will be made available by the authors, without undue reservation.
