# Supplementary material for: Normothermic ex vivo heart perfusion with NLRP3 inflammasome inhibitor Mcc950 treatment improves cardiac function of circulatory death hearts after transplantation
Source: Front Cardiovasc Med. 2023 Mar 17;10:1126391. doi: 10.3389/fcvm.2023.1126391 (PMC10063899; doi:10.3389/fcvm.2023.1126391)
Supplement: Supplementary file 1 [file Table1.docx]

Supplementary Material

## Operative Procedure

## Anesthesia

Rats were anesthetized with pentobarbital sodium (60 mg/kg, intraperitoneally). The pedal reflex was performed to determine adequate anesthetic depth before experiments. The rats were placed on a heating pad to maintain their body temperature.

## Harvest of Blood and Donor Heart from the Donor Rat

The rat was intubated with a 16 G, 2-inch I.V. catheter after tracheotomy and mechanical ventilation. A 22 G, 1-inch I.V. catheter was cannulated into the right carotid artery for the injection of heparin, real-time blood pressure monitoring, and delivery of Custodiol cardioplegic solution (Dr. Franz Köhler, Chemie GmbH, Bensheim, Germany). The injection of heparin (2,000 IU/kg body weight) through the right carotid artery was performed to heparinize the donor rat before the induction of circulatory death.

Rats were subjected to the following DCD procedure. An 18 G, 2-inch I.V. catheter was cannulated into the abdominal artery. A 20 ml syringe containing 1,250 IU heparin was connected to the catheter to withdraw blood as much as possible. Circulatory death was declared when systolic pressure below 30 mmHg or asystole was observed([1](#_ENREF_1), [2](#_ENREF_2)). Then 15-minute warm ischemia time (WIT, equivalent to the hands-off period) was allowed to elapse. The duration of warm ischemia time was from the declaration of circulatory death to the perfusion of donor hearts with the cold Custodiol cardioplegic solution.

At the end of the 15-minute WIT, we performed the sternotomy and clamped the aortic arch between the brachiocephalic trunk and the left common carotid artery. After cutting the inferior *vena cava*, the DCD heart was immersed with ice and perfused with 20 ml cold Custodiol cardioplegic solution at a constant pressure of between 60 and 80 mmHg for 5 min *via* the right carotid artery. The inferior and superior *vena cava*, and pulmonary veins were ligated and cut, then the left and right atrium were snipped. The pulmonary artery and the aorta distally to the left subclavian artery were cut, respectively. Subsequently, the arrested heart was excised and cannulated with a homemade 14 G aortic catheter. Finally, the donor heart was taken out of the chest cavity (Figure 2).

## The Operation of EVHP

The detailed normothermic EVHP protocol was described before([3](#_ENREF_3), [4](#_ENREF_4)).

The perfusion circuit consisted of a micro-peristaltic pump (BT101L; Lead Fluid; China), an oxygenator (Micro-1Rat Oxygenator; Dongguan Kewei; China), 16# Tygon tubing, a reservoir, an infusion syringe pump (Perfusor-space; B.Braun; Germany) for the administration of epinephrine (4.8 × 10−5 mg/h/kg body weight), and a home-made water-bath box containing water, heater, stirrer and temperature-controlling switch. The oxygenator was gassed with a humidified gas mixture containing 95% O_2_ and 5% CO_2_.

The perfusion circuit was primed with blood-based perfusate, which comprised of 8 ml blood from the rat, 6 ml modified Krebs–Henseleit solution (10 mM glucose, 117 mM NaCl, 4.5 mM KCl, 25 mM NaHCO_3_, 1.2 mM NaH_2_PO_4_, 2 mM CaCl_2_, 0.512 mM MgCl_2_), mannitol (25 g/L), methylprednisolone sodium succinate (500 mg/L; Pfizer; Belgium; Switzerland), and insulin (160 IU/L; Novo Nordisk; Denmark). The blood-based perfusate was slowly added into the perfusion circuit to be oxygenated for 15 minutes (PaO_2_ at 150–250 mmHg).

The reservoir was partially immersed in the water of the water-bath box and the isolated heart was placed below the horizontal plane of the water. The membrane oxygenator was wrapped by a thermal insulation bag and the temperature of water in the box was set at 41-42℃ to maintain the temperature of the inflow and the isolated heart at 35-37℃.

Briefly, the normothermic EVHP started with the perfusion flow rate of 1.8 ml/min, and slowly reached the target perfusion flow rate (10 ml/ (min·kg body weight)) within 10 min. The intraventricular pressure measurement of the donor heart was performed by inserting a balloon, which was connected to a pressure sensor, into the left ventricle through the left atrium and slowly filled the balloon with 0.10 ml saline at the end of 90-minute EVHP. The cardiac functional parameters of the donor heart during EVHP included developed pressure (DP, systolic blood pressure minus diastolic blood pressure), heart rate (HR), dP/dt_max_ (maximum rate of rise of left ventricular pressure), and dP/dt_min_ (maximum rate of pressure decline).

At the end of normothermic EVHP, the donor-heart was removed from the EVHP system, placed on ice, and perfused slowly with 5 ml cold Custodiol cardioplegic solution *via* the aortic catheter to remove the blood. Then the aorta distal to the brachiocephalic trunk was cut carefully and the notches of the left and right atrium were ligated with 4-0 nylon thread (Figure 2).

## Heterotopic Abdominal Heart Transplantation

As previously described([5](#_ENREF_5)), after anesthesia of the recipient rats, heterotopic abdominal heart transplantation was performed. Briefly, left external jugular vein cannulation was performed and 1 ml saline with a low dose of heparin (250 IU/kg) was administered. Both the abdominal artery and inferior *vena cava* were exposed and clamped by two delicate vessel clamps. 3 mm incisions were made in the abdominal artery and inferior *vena cava*.

End-to-side anastomosis was performed between the aorta and the abdominal aorta and between the pulmonary artery and the inferior vena cava with 8-0 prolene suture under the microscope. The vessel clamps were carefully released to reperfuse the donor heart after the completion of anastomosis. The end-to-side anastomosis should be completed in 40 minutes. Throughout the transplantation procedure, the donor heart was wrapped in moist gauze and 4℃ sterile saline was dropped on the surface of the donor heart every 5 minutes.

*In vivo* reperfusion lasted for 90 minutes after the donor heart was heterotopically transplanted into the recipient rat. Saline or saline with mcc950 was injected into the left external jugular vein 10 minutes after *in vivo* reperfusion. The rats were placed on the heating pad to keep their body temperature at 37℃. The measurement of left ventricular function and sample collection for histologic and molecular analyses were performed after 90-minute *in vivo* reperfusion. The intraventricular pressure was measured by inserting the balloon into the left ventricle through the heart apex and slowly filled the balloon with 0.10 ml saline.

## Sample Collection

All the heart specimen was taken at the end of the 90-minute *in vivo* reperfusion. Two ventricular slices (1 to 2 mm thick) were collected serially along the long axis of the graft. The first piece of myocardial tissue from the apex was preserved at -80℃ promptly and then applied for western blotting. The second one for immunofluorescence and immunohistochemical analysis was fixed in paraformaldehyde solution (4%), embedded in paraffin, and finally cut into a 4-µm-thick section.

The blood samples from the recipient rats were drawn from the abdominal aorta with an 18 G, 2-inch I.V. catheter. Then, plasma samples were obtained after centrifugation (3000 rpm, 15 min, 4 °C).

Reference

1. Korkmaz-Icoz S, Li K, Loganathan S, Ding Q, Ruppert M, Radovits T, et al. Brain-dead donor heart conservation with a preservation solution supplemented by a conditioned medium from mesenchymal stem cells improves graft contractility after transplantation. American journal of transplantation : official journal of the American Society of Transplantation and the American Society of Transplant Surgeons. 2020;20(10):2847-56.

2. Zhou P, Liu H, Liu X, Ling X, Xiao Z, Zhu P, et al. Donor heart preservation with hypoxic-conditioned medium-derived from bone marrow mesenchymal stem cells improves cardiac function in a heart transplantation model. Stem cell research & therapy. 2021;12(1):56.

3. Li J, Xue C, Ling X, Xie Y, Pavan D, Chen H, et al. A Novel Rat Model of Cardiac Donation After Circulatory Death Combined With Normothermic ex situ Heart Perfusion. Frontiers in cardiovascular medicine. 2021;8:639701.

4. Lu J, Xu L, Zeng Z, Xue C, Li J, Chen X, et al. Normothermic ex vivo Heart Perfusion Combined With Melatonin Enhances Myocardial Protection in Rat Donation After Circulatory Death Hearts via Inhibiting NLRP3 Inflammasome-Mediated Pyroptosis. Frontiers in cell and developmental biology. 2021;9:733183.

5. Takahashi M. Cell-Specific Roles of NLRP3 Inflammasome in Myocardial Infarction. Journal of cardiovascular pharmacology. 2019;74(3):188-93.
